# Metaverse surgical planning for robotic surgery: preliminary experience and users’ perception

**DOI:** 10.1177/17562872241297524

**Published:** 2024-12-16

**Authors:** Gabriele Volpi, Cecilia Gatti, Alberto Quarà, Federico Piramide, Daniele Amparore, Paolo Alessio, Sabrina De Cillis, Marco Colombo, Giovanni Busacca, Michele Sica, Paolo Verri, Alberto Piana, Stefano Alba, Michele Di Dio, Cristian Fiori, Francesco Porpiglia, Enrico Checcucci

**Affiliations:** Department of Surgery, Candiolo Cancer Institute, FPO-IRCCS, Strada Provinciale 142, km 3.95 - 10060 Candiolo, Turin, Italy; Department of Surgery, Candiolo Cancer Institute, FPO-IRCCS, Candiolo, Turin, Italy; Department of Oncology, Division of Urology, University of Turin, San Luigi Gonzaga Hospital, Orbassano (TO), Piemonte, Italy; Department of Oncology, Division of Urology, University of Turin, San Luigi Gonzaga Hospital, Orbassano (TO), Piemonte, Italy; Department of Oncology, Division of Urology, University of Turin, San Luigi Gonzaga Hospital, Orbassano (TO), Piemonte, Italy; Department of Surgery, Candiolo Cancer Institute, FPO-IRCCS, Candiolo, Turin, Italy; Department of Oncology, Division of Urology, University of Turin, San Luigi Gonzaga Hospital, Orbassano (TO), Piemonte, Italy; Department of Oncology, Division of Urology, University of Turin, San Luigi Gonzaga Hospital, Orbassano (TO), Piemonte, Italy; Department of Oncology, Division of Urology, University of Turin, San Luigi Gonzaga Hospital, Orbassano (TO), Piemonte, Italy; Department of Oncology, Division of Urology, University of Turin, San Luigi Gonzaga Hospital, Orbassano (TO), Piemonte, Italy; Department of Oncology, Division of Urology, University of Turin, San Luigi Gonzaga Hospital, Orbassano (TO), Piemonte, Italy; Romolo Hospital, Rocca di Neto (KR), Italy; Division of Urology, Department of Surgery, SS Annunziata Hospital, Cosenza, Italy; Division of Urology, Department of Surgery, SS Annunziata Hospital, Cosenza, Italy; Division of Urology, Department of Surgery, SS Annunziata Hospital, Cosenza, Italy; Department of Oncology, Division of Urology, University of Turin, San Luigi Gonzaga Hospital, Orbassano (TO), Piemonte, Italy; Department of Oncology, Division of Urology, University of Turin, San Luigi Gonzaga Hospital, Orbassano (TO), Piemonte, Italy; Department of Surgery, Candiolo Cancer Institute, FPO-IRCCS, Candiolo, Turin, Italy

**Keywords:** 3D models, kidney cancer, metaverse, new technologies, prostate cancer, robotic surgery

## Abstract

**Background:**

The 3D models’ use for surgical planning has recently gained an ever-wider popularity, in particular in the urological field. Different ways of fruition of this technology have been evaluated over the years. Today, new technological developments allow us to enjoy 3D models in the metaverse.

**Objectives:**

The aim of this study is to report the preliminary experience and surgeon’s perception of preoperative planning performed in the metaverse.

**Design:**

During the eleventh edition of the Techno-Urology Meeting, all the attendees enjoyed the metaverse experience (META_EXP) for pre-surgical planning of both robot-assisted radical prostatectomy and partial nephrectomy. Users’ perception was then evaluated with the Health Information Technology Usability Evaluation Scale (Health-ITUES) and the Face & Content validity questionnaire.

**Methods:**

The 3D virtual models, obtained from standard bi-dimensional imaging, were uploaded on a metaverse platform. Surgeons, thanks to dedicated visors, could plan their surgical strategy immersed in this virtual environment and discuss it with other attendees. Answers to the questionnaires were then evaluated and a stratification was subsequently performed based on surgical expertise, dividing participants in residents (Re), young urologists (YU) and senior urologists (SU).

**Results:**

Sixty-six participants filled out the questionnaires. As emerged from the Health-ITUES questionnaire, META_EXP covers an important role in the presurgical/surgical planning and decision-making process and appears to be useful for preoperative planning, with a median response of 4 and 5, respectively. Such results were also confirmed at the Face & Content validity questionnaire, with a median rate of 9/10 regarding its usefulness for surgical planning. Also, anatomical accuracy was positively rated regarding both organ’s and disease’s details, with a median response of 9.

**Conclusion:**

In conclusion, the metaverse experience for preoperative surgical planning appears to be useful, user-friendly and accurate. This technology has been widely appreciated by surgeons, irrespective of their experience.

## Introduction

Over the last lustrum, 3D models’ use—whether printed or enjoyed on a digital platform—for the surgical planning of both robot-assisted radical prostatectomy (RARP) and partial nephrectomy (RAPN) has gained an ever-wider popularity among urologists.^1^ In fact, these reconstructions allow a deeper and more precise understanding of patients’ anatomical details and have therefore allowed to enter in the ‘precision surgery era’.^2^ Concerning RARP, 3D models are particularly relevant in the highlighting of potential tumour capsular contact or extra-capsular extension (ECE), giving surgeons the opportunity to specifically tailor the nerve sparing phase of the procedure while potentially reducing the incidence of positive surgical margins.^3^ Regarding RAPN, the impact of this technology is even more relevant since it allows to better appreciate lesion’s location in case of endophytic lesions and the possible contact with intraparenchymal structures such as vessels and calyces.^4^ Moreover, 3D models’ use in the planning of RAPN improves surgeon’s comprehension of kidney’s vascular anatomy, which is highly variable from one patient to another. The 3D reconstructions today available have even pushed the boundaries of this concept, thanks to the integration of mathematical algorithms, and are able to show the different perfusion zones of the kidney, allowing surgeons to simulate the clamping strategy during the surgical planning.^5^

The 3D models’ use for preoperative planning can be exploited in different fashions. First, such models can be used in a 3D printed cognitive setting, in which the virtual reconstructions are physically printed and evaluated before the surgical procedure by the surgeons.^6^ Another option consists in their use in a virtual cognitive setting, in which models are made available on a digital support (tablet, PC), and surgeons can rotate them and modify the transparency of the different anatomical structures.^7^ Furthermore, they can also be enjoyed in a mixed reality setting, thanks to the use of head mounted display systems (i.e. Hololens), giving surgeons the opportunity to ‘walk around’ the model, evaluating it from different perspectives, interacting with them thanks to hand gestures.^8^

Thanks to further technological developments such as a wider adoption of 5G internet connection and a general improvement of digital literacy, boosted by the Covid-19 pandemic and its restrictions, the use of a new technological tool is today emerging in urology: the metaverse.^9,10^ which is considered as the next iteration of the internet and refers to a virtual space in which users can interact with digital contents and other users. This concept has aroused increasing attention in various fields such as social networks, e-commerce, gaming and recreation. However, one of the most fascinating fields of application is represented by the healthcare, in which the potentials of this emerging technology are widely unexplored and still under scrutiny. In this domain, one of its potential roles lies precisely in the improvement of preoperative planning. In fact, 3D reconstructions can be uploaded in the metaverse and, thanks to specific visors, enjoyed in a completely immersive reality.

Multiple applications of virtual reality in urology were reported in pre-operative surgical planning for kidney and prostate tumours.^7,11,12^ The advent of the metaverse allowed to carry out some pioneering experiences, letting experts evaluate and discuss complex cases, remotely.^22^

Herein we explored the metaverse surgical planning before RARP and RAPN: in such immersive virtual world surgeons were able to interact with the 3D models and fully apprehend anatomical details of every single case, planning their surgical strategy on a ‘tailored’ basis. Subsequently, we evaluated surgeons’ feedback using validated questionnaires.

## Materials and methods

### Objectives

The objectives of this study are to report our preliminary experience with the application of metaverse surgical planning with 3D virtual models and to assess its usability and Face & Content validity.

The reporting of this study conforms to the Strengthening the Reporting of Observational Studies in Epidemiology (STROBE) statement.^13^

### Study design

The metaverse approach to surgical planning (META_EXP) has been tested during the eleventh edition of the Techno Urology Meeting (TUM), a live-surgery congress, held in the Hospitals IRCCS Candiolo and San Luigi Gonzaga in Turin between the 13th and 14th of April 2023.

In this study, all the participants (surgeons, moderators and attendees) to the eleventh TUM had the possibility to evaluate the META_EXP. Before the execution of every single live-surgery procedure, surgeons and moderators wore dedicated visors that allowed them to access the metaverse. The whole experience was broadcasted on screens in the auditorium, engaging also the attendees. Once entered in the digital environment, both surgeons’ and moderators had the possibility to interact with the 3D reconstructions specifically created for every single patient, highlighting anatomical and tumours’ details. Surgeons could then evaluate and explain their surgical strategy, on a ‘tailored’ basis, and discuss it with the moderators and the audience. Physical distance, due to the location of the operators in different hospitals, was then virtually abolished, with surgeons’ and moderators’ avatars gathering around the virtual reconstruction of the organ in the metaverse and debating of the surgical procedure. Once the surgical planning in the metaverse was discussed with the audience the metaverse experience came to an end, surgeons moved to the operating room and performed the procedures, which were broadcasted in the auditorium.

Furthermore, close to the auditorium, in a dedicated space, the attendees had the opportunity to enjoy firsthand the metaverse experience wearing the visors, interacting and navigating the 3D models as surgeons and moderator did, in the digital environment.

At the end of their experience, participants were asked to fill out two dedicated questionnaires assessing the usability and validity of the metaverse in the planning of robotic surgery. According to their level of expertise, surgeons were classified in residents (Re), young urologists (YU), senior urologists (SU; >40 years old), and on the basis of their surgical skills (open, laparoscopic, robotic). Written consent and IRB approval were not required for the present study.

### 3D models’ production and upload on the metaverse

Standard preoperative assessment for both RARP and RAPN still relies on standard bi-dimensional imaging, such as CT scan or MRI. However, in particular for Re and YU, the interpretation and full comprehension of patients’ and tumours’ anatomies based only on these investigations might be suboptimal, due to a demanding ‘building in mind process’. As mentioned above, 3D reconstructions provide easily accessible information, thanks to their spatial definition.

Time ago, 3D reconstructions were obtained from common DICOM format viewer software, of which most radiology departments are equipped with. However, such reconstructions were far from ideal, with poor anatomical details and, frequently, the inability to distinguish the various portions of the organs (arteries, veins, parenchyma, tumoural lesion).

Today, 3D models’ production is based on a multidisciplinary teamwork, in which urologists, radiologists and bioengineers are all involved with their different skills and expertise.^14^ Our group collaborates with a company named Medics Srl (https://www.medics3d.com/) for 3D models’ production. The first step of models’ production still is represented by the execution of bi-dimensional imaging investigations (CT or MRI), in which single’s slice thickness should be lower than 5 mm in order to increase reproduction’s quality. Images are then exported in DICOM format.

In the ‘preprocessing’ phase, thanks to a dedicated DICOM visualisation software (*MicroDicom viewer*), the target of interest is thoroughly inspected, extrapolating the most appropriate images (arterial phase for CT scans and T2 sequences in case of MRI) and varying some image parameters such as contrast, brightness and luminance. Subsequently, a volume rendering of the organ is automatically obtained, using data from the picture voxels. Then, with a dedicated software called Mimics Medical 21.0 (Materialise, Leuven, Belgium), the ‘segmentation’ phase begins, in which pixels belonging to a specific region of interest (ROI) are separated from surrounding structures. The technique chosen for ROIs’ distinction is called ‘thresholding’, involving the selection of a specific range of a defined parameter (e.g. grey scale). Once the range is set, the software identifies regions with the specified characteristics, automatically discarding other regions/objects. During this phase, the software may fail in the representation of some anatomical details and a manual intervention by dedicated bio-engineers could be required.

The model can then be exported and saved in.stl (Standard Triangulation Language) format. In particular, each anatomical part is saved in .stl format, so there are as many 3D objects in STL (stereolithography) format as the anatomical parts you want to represent. Subsequently, the STLs are imported into a proprietary software that assigns a colour to each object and generates the PDF3D. Some modifications to the rendering are still possible at this phase using the software 3-matic Medical 13.0 (Materialise, Leuven, Belgium). After this step, the 3D virtual reconstruction is completed and ready for its use on the different electronic devices.

**Figure 1.**
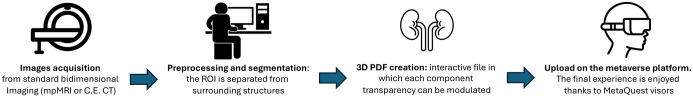
Diagram describing the process of metaverse technology implementation.

**Figure 2.**
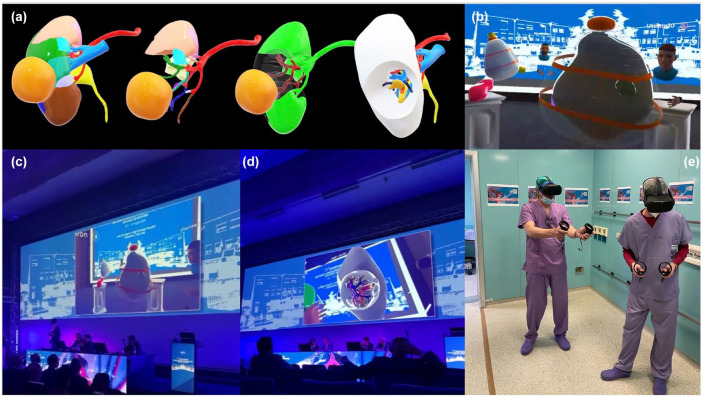
Meta_EXP: (a) 3D virtual phantoms models for kidney sparing surgery, (b) 3D model of the prostate and prostate cancer (green) visualised into the metaverse, (c) and (d) the Meta_Exp was enjoyed by the whole auditorium during TUM23 edition, and (e) the surgeon wore the virtual reality visor to navigate the model into the metaverse.

Specifically for the eleventh TUM, the 3D virtual reconstructions obtained were then uploaded on a metaverse platform developed by *Anothereality* (https://www.anothereality.io), which creates an immersive environment and a virtual experience in a complete cloud platform. The access to this virtual environment is ensured by *MetaQuest* visors, virtual reality head-mounted displays produced by *Meta*, letting users enter in a completely immersive virtual world in which the user is embodied by an avatar capable of interaction thanks to specific controllers (Figure 1). Regarding kidney models, four different pre-sets were created aiming to show model’s role during surgery: the first one showed the kidney with its different vascular regions and its various colours on organ’s surface, the second one highlighted the vascular regions contacting the tumour, the third one the suggested selective clamping strategy and the fourth one the tumour’s resection bed (Figure 2(a) and (d)). Concerning prostate models, the tumoural lesion and its potential capsular contact were highlighted: surgeons could then modify the transparency of the different structures in order to better apprehend the surgical anatomy (Figure 2(b) and (c)). All the gestures were performed thanks to dedicated joysticks (Figure 2(e)).

### Health-ITUES and Face & Content validity questionnaires

As mentioned above, all the participants to the eleventh TUM congress were asked to fill out two specific questionnaires, reported in Supplemental Material (Annex 1), evaluating their impressions and experience with the metaverse surgical planning.

The first questionnaire is the Health-ITUES (health information technology usability evaluation scale), divided in 24 questions (Q5–Q24) and assessing mobile health technologies.^15^ It is based on an integrated model of multiple usability theories built on the concepts of usability from the Technology Acceptance Model^16^ and the International Organization for Standardization (ISO) standard 9241-11.^17^ The questionnaire is divided in four subparts evaluating the overall impact, the usefulness, the ease of use and users’ control of the technology in question. The main advantage of this questionnaire is represented by the fact that it is fully customisable, without the need for item addition, deletion or modification.

The second questionnaire was purpose-built for Face and Content validity investigation (Q1a-QSc).^16^ Also, this questionnaire was divided in four subparts analysing the overall usefulness, the anatomical accuracy, the utility in the surgical planning and new potential implication of this technology. The questions were open-ended with answers based on ordinal ten-point rating Likert scales (where 1 was strongly no and 10 was definitely yes). Subjective questions for Face validity (Q1 and Q3) or objective questions (Q2 a, b and S a, b, c, d) for content validity were included. Content validity was tested using the content validity ratio.

### Statistical analysis

Descriptive statistics includes frequencies and proportions. Differences between the answers of the three different groups of participants (Re, YU, SU) were assessed using the Kruskal–Wallis test. All the analysis were performed using the Jamovi software (v. 2.3, Sydney, Australia). Statistical significance was set as *p* < 0.05.

## Results

### Study population

Sixty-six participants responded to the two questionnaires. Among them, 20 (33.3%) were females. Considering the surgical experience, 42 (63.7%) were residents, 16 (24.2%) were YU and 8 (12.1%) SU. Most of them performed mini-invasive surgery and, in particular, robotics (48, 72.7%). See Table 1.

**Table 1.** Population description of the attendees that enjoy Meta_EXP and filled out the questionnaire.

Variable	Overall participants (*n* = 66)
Gender, *n* (%)	
Male	46 (67.7)
Female	20 (33.3)
Level of expertise, *n* (%)	
Residents	42 (63.7)
Young urologist	16 (24.2)
Senior urologist	8 (12.1)
Surgical field of expertise, *n* (%)	
Open surgery	32 (48.5)
Laparoscopic surgery	30 (45.5)
Robotic surgery	48 (72.7)

### Health-ITUES questionnaire

**Figure 3.**
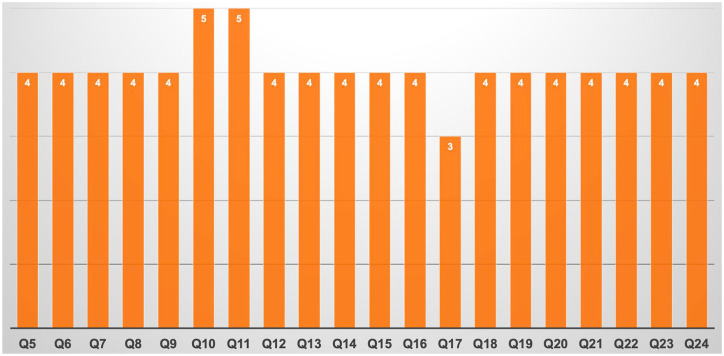
Median answers to the Health-ITUES questionnaire. Health-ITUES, Health Information Technology Usability Evaluation Scale.

Concerning the overall impact of the Meta_EXP, it revealed to have an added value for surgeons, in particular concerning the presurgical/surgical planning and the intraoperative decision-making, with 87.9% and 72.7% of the responders who answered 4 (agree) or 5 (strongly agree) to the questions 6 and 7, respectively (Figure 3). Moreover, also the potential added value for patients was positively rated, with a median response of 4 (agree, Q5).

Regarding the ‘Perceived usefulness’ (Q8–Q16) the median response was 4 (agree) for every question, except for a peak of 5 (strongly agree) in median response registered for Q10 (IQR 4–5), comparing this technology to standard bi-dimensional imaging, and Q11 (IQR 4–5), evaluating the usefulness of Meta_EXP for pre-surgical planning.

As regards the ‘Perceived ease of use’, the median response to Q17, investigating the comfort in the ability to use this technology, was three (IQR 3–5), while the median response was four for questions Q18–Q21, investigating in particular the ability to become skilful with such technology and Meta_EXP’s simplicity of use.

Finally, assessing the ‘User control’, the ease of comprehension of error messages (Q22) was scored >4 for 53.7% of the responders; the possibility to recover from an error (Q23) was rated >4 for 57.6% of the attendees, and the clarity of information displayed was rated > 4 in 66.7% of the cases.

### Face & Content validity questionnaire

**Figure 4.**
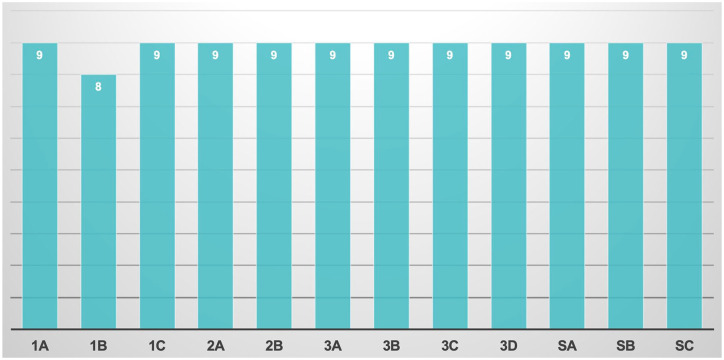
Median answers to the Face & Content validity questionnaire.

Regarding the median evaluation of usefulness of Meta-EXP (Q1a), it was rated 9/10 (IQR 7–9) (Figure 4). Similarly, also its potential added value compared to standard bi-dimensional imaging (Q1b (IQR 7–9)) and its usefulness for case discussion with patients (Q1c (IQR 8–10)) was scored 9/10. Concerning the anatomical accuracy of 3D models displayed in the metaverse to reproduce both the organ (Q2a) and the disease (Q2b), it was rated >9 in 57.6% of the cases.

As regards the usefulness in surgical planning (Q3a) the median response was 9/10 (IQR 8–9). In addition, also the role in the apprehension of surgical complexity (Q3b (IQR 8–10)) and the potential added value in oncological efficacy and functional outcomes (Q3c (IQR 7–10)) were rated 9/10. The potential role in surgical training (Q3d) was rated > 9/10 for 75.8% of the attendees.

Regarding specific questions, the median evaluation of Meta-EXP’s capability to improve patients’ counselling was rated 9/10 (QSa (IQR 8–10)). Moreover, also the possibility to improve the quality of surgical case discussion and preoperative planning thanks to the possibility of bringing together the various experts in a single virtual room was rated 9/10 (QSa (IQR 8–10)). Finally, its possible implication in virtual lessons and surgical training reached the same score (QSc (IQR 9–10)).

### Answers’ evaluation based on surgical expertise

**Figure 5.**
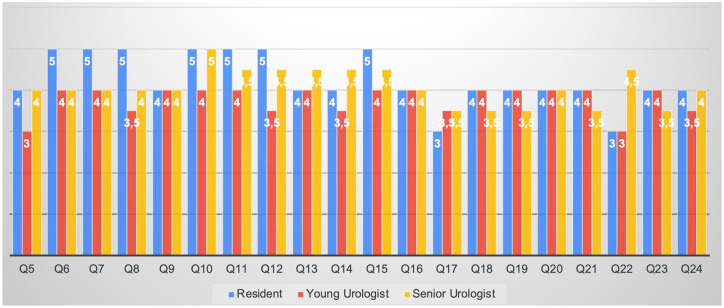
Median answers to the Health-ITUES questionnaire stratified by level of expertise. Health-ITUES, Health Information Technology Usability Evaluation Scale.

Concerning the Health-ITUES questionnaire, all the three groups defined the META-EXP as a positive addition for surgeons (Q6), with a median response of 4 for YU and SU and a peak of 5 for Re (*p* = 0.46). Similarly, the role in pre-surgical planning (Q7) was rated 5 by Re and 4 by more experienced urologists (YU and SU) (*p* = 0.19) (Figure 5).

The ‘perceived usefulness’ of META_EXP corroborated these findings, with attendees evaluating it as a useful tool in order to improve speed and quality of the surgical planning (Q9, Q11, Q15), exceeding bi-dimensional imaging (Q10), without significant difference between three groups (Q9 *p* = 0.41; Q11 *p* = 0.23; Q15 *p* = 0.12; Q10 *p* = 0.36).

Concerning the ‘ease of use’ part of the questionnaire, considering the short time of manipulation, attendees evaluated their ability to use META_EXP > 4 in 47.6%, 50% and 50% of the cases for Re, YU and EU (Q17 *p* = 0.98). Moreover, the simplicity of use was rated >4 by 52.4%, 77% and 74% of Re, YU and SU, respectively (Q20 *p* = 0.67).

Regarding ‘user control’, the possibility to recover from an error was evaluated >4 by 57.1%, 75.6% and 50% of Re, YU and SU, respectively (Q23 *p* = 0.47).

As regards the Face & Content validity questionnaire, the three categories agreed concerning the usefulness of META_EXP with a median response of 8, 9 and 9.5 for Re, YU and SU, respectively (Q1a *p* = 0.32). The results were astonishing for all the three categories about anatomical accuracy. This is the only case in which YU and SU evaluation even exceeded, in a statistically significant manner, RE’s evaluation for organ’s details with a median response of respectively 9, 10 and 8 for organ’s details and of, respectively, 9.5, 9.5 and 8 (Q2a *p* = 0.015; Q2b *p* = 0.049).

**Figure 6.**
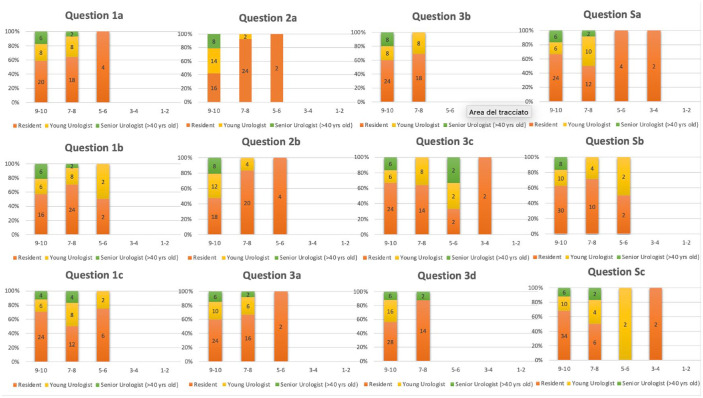
Answers to the Face & Content validity questionnaire stratified by level of expertise.

With regard to the potential usefulness of META_EXP for surgical planning, the judgement was extremely positive, with responses >9 in 57.1%, 62.5% and 75% of Re, YU and SU, respectively (Q3a *p* = 0.51) (Figure 6). Moreover, the potential of such technology to provide information regarding the surgical complexity was rated >9 in 57.1%, 50% and 100% of Re, YU and SU, respectively (Q3a *p* = 0.35). A potential application in surgical training was also positively rated, with evaluations >9 in 66.7%, 100% and 75% of Re, YU and SU, respectively (Q3a *p* = 0.35). Finally, the median evaluation of the potential improvement of surgical case discussion and preoperative planning was 9, 9 and 10 for Re, YU and SU, respectively (QSb *p* = 0.12).

## Discussion

In this study, we introduce a newly developed Meta_EXP designed specifically for 3D model-guided surgical planning in RARP and RAPN. This is the first time that usability and users’ perception of this platform are being presented and evaluated.

The concept of metaverse has recently gained significant attention, even within the healthcare domain. In essence, the metaverse is a derivative of the internet of medical things, which involve the integration of physical objects into the internet. This integration enables perception, transmission, and intelligent processing in various settings. To put it simply, the metaverse represents the next evolution of the internet, incorporating artificial intelligence, augmented reality, virtual reality (VR) and advanced network connectivity like 5G. These technologies, in essence, work together to create immersive and interactive online environments.^9,10,18,19^

Regarding the Health-ITUES questionnaire, the Meta_EXP demonstrated to have a positive additive value for both patients and surgeons with 60.6% and 90.9% of the attendees who responded 4 (agree) or 5 (strongly agree) to Q5 and Q6, respectively. Furthermore, it is interesting to denote that the perceived usefulness questions were again scored with a median score of 4, with a peak of 5 for the questions regarding the surgical planning (Q10 and Q11).

Moreover, it is worth mentioning that, despite the stratification on the basis of surgical expertise, the questions evaluating the usefulness of Metaverse platform showed good results across the three groups (Re, YU, SU). In particular, the perceived ease of use was well appreciated by all the groups.

The Meta_EXP was also evaluated using our previously validated Face and Content validity questionnaire, specifically developed for 3D models guided surgery.

The findings of our research demonstrate the potential of this new technology in the field of urology. Promising outcomes were observed in terms of effectiveness for surgical planning, with a median response of 9 out of 10 to all the questions of the dedicated part of the questionnaire (3a, 3b, 3c, 3d). Also concerning anatomical accuracy this technology showed impressive results, with scores >8 in 93.9% and 90.9% of the cases for questions 2a and 2b, respectively. Interestingly, only for these two questions, a statistically significant difference was recorded in favour of more experienced surgeons (YU and SU) compared to Re, as previously mentioned. Furthermore, the improved anatomical apprehension and the high quality of the models seem to have a potential benefit also for patients counselling (Sa, scored 9/10).

Our Meta_EXP is perfectly integrated in the current concept of technology-driven surgery. It represents the latest advancement in 3D model-guided surgery, which our team has been developing since 2018.^1^

The core focus of Meta_EXP lies precisely in the use of 3D models. As outlined in the Materials and methods section, these models have undergone significant advancements. In fact, regarding kidney cancer models, we have enabled navigation and modulation of transparency for various anatomical structures within the phantoms. Moreover, these models now incorporate functional information, such as detailed perfusion areas. Consequently, as we have recently demonstrated, it is now possible to predict the specific perfusion zones supplied by every single artery. This breakthrough enables precise planning of clamping techniques and simulation of the ischaemic effects on the parenchyma.^5,20^

Similarly, concerning prostate cancer, the external portion of the tumour, with the suspicious extra-capsular contact, can be clearly visualised and highlighted into the model, tailoring the nerve sparing phase and potentially reducing positive surgical margins.^3^

Speaking about our newly introduced technology, the metaverse experience, we recently demonstrated how this new kind of visualisation and interaction can allow to overcome, or at least mitigate, physical barriers.^21^ In fact, compared to other VR visualisation modalities, such as mixed reality, in which virtual content can be experienced by users in the same physical space, the metaverse combines a virtual immersive reality with the capabilities of high-speed internet connection. As a result, virtual content can then be shared remotely, bringing users together in the same virtual space, regardless of their physical locations. This breakthrough in connectivity enables a truly immersive and collaborative experience within the metaverse.

The good scores obtained with our Meta_EXP evaluation by these two validated questionnaires reflect the preliminary findings that we recently reported especially for preoperative surgical planning of mini-invasive partial nephrectomy within the metaverse.^21^ The surgeons, thanks to the presence of their avatar into the virtual space of metaverse, had a great sense of ‘being there’, and could plan their surgeries sharing the surgical strategy with other colleagues physically located in different hospitals. This technology appears to have multiple advantages and a wider implementation in the future could have positive implications in the surgical ‘routine’. In fact, the anatomical apprehension of both the organ and the tumour were greater compared to standard bi-dimensional imaging. The technology was particularly useful for surgical planning, allowing to carry it out in a precise and timely manner, providing also some additional information regarding the complexity of every single case. This could then have some positive repercussions on surgical outcomes, both functional and oncological.

Moreover, this new way of fruition of 3D models offers the opportunity to share the personal experience and can have a great role in the reduction of physical distances, allowing the execution of ‘high-level’ consultations even in remote areas of the world. The impact can then be relevant for both patients and surgeons, improving the quality of medical assistance.

However, as it often happens when a new technology is introduced, some drawbacks should be considered. First, the excessive application of Metaverse in surgical planning can present risks like overestimated accuracy (virtual models might lead to overconfidence, potentially missing real-world anatomical variations); skill degradation (excessive use could reduce hands-on skills and manual dexterity) and technical failures (dependence on technology introduces risks of glitches and malfunctions). Furthermore, the Metaverse, while offering vast opportunities for innovation and interaction, raises several ethical concerns. Privacy and data security are paramount, as users’ personal information and behaviours are tracked and stored, posing risks of data breaches and unauthorised surveillance. Then, identity and representation become complex, with possibilities of identity theft and the creation of misleading or harmful avatars. Digital addiction is another critical issue, as the immersive nature of the Metaverse can lead to excessive use, impacting mental health and real-world responsibilities. Additionally, this technology might exacerbate inequalities, as access to advanced technology is not universal. In fact, the costs for the implementation of this whole technology, from images acquisition to 3D models’ creation and upload on a metaverse platform are not negligible. Finally, content regulation is challenging, with potential for the spread of misinformation, cyberbullying and inappropriate content. Addressing these ethical issues is essential to create a safe and inclusive Metaverse environment.

Moreover, despite the wide appreciation expressed by surgeons, the real clinical benefit determined by the implementation of this technology still needs to be evaluated in further studies. Finally, our study population is limited, and future works should focus on larger samples to further validate our findings with more statistical power.

In the next future, a larger diffusion of 5G connection, the decrease of costs of head-mounted display systems and the improvement of dedicated platforms for medical use will bring to a larger diffusion of the Metaverse experience, potentially expanding the knowledge sharing and the quality of surgical planning. In particular, the creation of a dedicated platform will allow to create and share into the VR the 3D models, and multi-center prospective studies are recommended to further validate the usefulness of such technology.

## Conclusion

The Metaverse experience for pre-operative surgical planning represents a new immersive tool able to gather physically distant surgeons in the same virtual environment. As emerged from the impressive results obtained on both Health-ITUES and Face & Content validity questionnaires, this new technology appears to be useful, user-friendly and accurate, exceeding the limits of standard bi-dimensional imaging. The appreciation was expressed by all the three categories of attendees, notwithstanding their different level of expertise. However, our study population is limited and only 12.1% was represented by SU: further studies should be encouraged in order to evaluate ‘older’ urologists’ judgement of this technology.

## Supplemental Material

sj-docx-2-tau-10.1177_17562872241297524 – Supplemental material for Metaverse surgical planning for robotic surgery: preliminary experience and users’ perception Supplemental material, sj-docx-2-tau-10.1177_17562872241297524 for Metaverse surgical planning for robotic surgery: preliminary experience and users’ perception by Gabriele Volpi, Cecilia Gatti, Alberto Quarà, Federico Piramide, Daniele Amparore, Paolo Alessio, Sabrina De Cillis, Marco Colombo, Giovanni Busacca, Michele Sica, Paolo Verri, Alberto Piana, Stefano Alba, Michele Di Dio, Cristian Fiori, Francesco Porpiglia and Enrico Checcucci in Therapeutic Advances in Urology

sj-pdf-1-tau-10.1177_17562872241297524 – Supplemental material for Metaverse surgical planning for robotic surgery: preliminary experience and users’ perception Supplemental material, sj-pdf-1-tau-10.1177_17562872241297524 for Metaverse surgical planning for robotic surgery: preliminary experience and users’ perception by Gabriele Volpi, Cecilia Gatti, Alberto Quarà, Federico Piramide, Daniele Amparore, Paolo Alessio, Sabrina De Cillis, Marco Colombo, Giovanni Busacca, Michele Sica, Paolo Verri, Alberto Piana, Stefano Alba, Michele Di Dio, Cristian Fiori, Francesco Porpiglia and Enrico Checcucci in Therapeutic Advances in Urology

## Declarations

**Ethics approval and consent to participate:** According to Italian law (Agenzia Italian del Farmaco guidelines for observational studies, 20 March 2008) no formal institutional review or ethics committee approval was required.

**Consent for publication:** Not applicable.

**Availability of data and materials:** Not applicable.

**Patient consent:** All the patients signed informed consent for participation in this study.
