# Supplementary material for: Metaverse surgical planning for robotic surgery: preliminary experience and users’ perception
Source: Ther Adv Urol. 2024 Dec 16;16:17562872241297524. doi: 10.1177/17562872241297524 (PMC11648019; doi:10.1177/17562872241297524)
Supplement: sj-docx-2-tau-10.1177_17562872241297524 – Supplemental material for Metaverse surgical planning for robotic surgery: preliminary experience and users’ perception [file sj-docx-2-tau-10.1177_17562872241297524.docx]

**11^TH^ TECHNO-UROLOGY MEETING (TUM) – 13-14 APRIL 2023**

**METAVERSE EXPERIENCE (META_EXP) EVALUATION**

1. Country: _____________________________________
2. Gender: Male Female
3. Position: Resident Young Urologist Senior Urologist (>40 yrs old)
4. Surgical skill (MCQ):

Open Laparoscopic Robotic

**Health Information Technology Usability Evaluation Scale (5 strongly agree – 1 strongly disagree)**

**Impact:**

1. I think META_EXP has been a positive addition for the patient. 1_2_3_4_5
2. I think META_EXP has been a positive addition for the surgeon. 1_2_3_4_5
3. META_EXP covers an important role in my presurgical/surgical planning and decision making process. 1_2_3_4_5

**Perceived usefulness**

1. The use of META_EXP makes it easier to understand the patient’s and disease’s anatomy. 1_2_3_4_5
2. The use of META_EXP enables me to plan my surgical strategy more quickly. 1_2_3_4_5
3. The use of META_EXP makes it more likely for me to rely on standard bidimensional imaging (CT/MRI). 1_2_3_4_5
4. META_EXP is useful for surgical planning. 1_2_3_4_5
5. I think META_EXP presents a more equitable process for surgical strategy. 1_2_3_4_5
6. I am satisfied with the use of META_EXP for surgical strategy decision-making process (both planning and simulation). 1_2_3_4_5
7. I can consult and study the clinical case in a timely manner because of META_EXP. 1_2_3_4_5
8. Using META_EXP increases the quality (in terms of precision and safety) of my surgical planning and subsequent intervention. 1_2_3_4_5
9. I am able to self-manage surgical planning and navigation whenever I use META_EXP. 1_2_3_4_5

**Perceived ease of use**

1. I am comfortable with my ability to use META_EXP. 1_2_3_4_5
2. Learning to work with META_EXP is easy for me. 1_2_3_4_5
3. It is easy for me to become skillful at using META_EXP. 1_2_3_4_5
4. I find META_EXP easy to use. 1_2_3_4_5
5. I can always remember how to log on to and use META_EXP. 1_2_3_4_5

**User control**

1. META_EXP gives error messages that clearly tell me how to fix problems. 1_2_3_4_5
2. Whenever I make a mistake using META_EXP, I recover easily and quickly. 1_2_3_4_5
3. The information (such as on-line help, on-screen messages, and other documentation) provided with META_EXP is clear. 1_2_3_4_5

**Face and Content Validity Questionnnaire (1 is strongly no** and **10 is definitely yes)**

**Q.1: OVERALL USEFULNESS**

1a) How do you evaluate the usefulness of the META_EXP? 1_2_3_4_5_6_7_8_9_10

1b) Do you think the META_EXP models can offer additional information with respect to the gold standard imaging used in diagnosis and local staging of the disease before the surgery? 1_2_3_4_5_6_7_8_9_10

1c) Do you think the META_EXP could be useful to facilitate the case discussion with the patient? 1_2_3_4_5_6_7_8_9_10

**Q.2: ANATOMICAL ACCURACY**

2a) How do you evaluate the accuracy of 3D models in META_EXP in reproducing the anatomical details of the organ? 1_2_3_4_5_6_7_8_9_10

2b) How do you evaluate the accuracy of 3D META_EXP models in reproducing the anatomical details of the disease? 1_2_3_4_5_6_7_8_9_10

**Q.3: SURGICAL PLANNING**

3a) How do you evaluate the usefulness of the META_EXP in the surgical planning? 1_2_3_4_5_6_7_8_9_10

3b) Do you think that the META_EXP could offer additional information in understanding the surgical complexity? 1_2_3_4_5_6_7_8_9_10

3c) Do you think that the META_EXP could increase the oncological efficacy and the functional outcomes after surgery? 1_2_3_4_5_6_7_8_9_10

3d) How do you evaluate the usefulness of the META_EXP in surgical training? 1_2_3_4_5_6_7_8_9_10

**Q.S: SPECIFIC QUESTIONS**

Sa) Do you think that the META_EXP could improve the quality of patient’s counseling thanks to the possibility of bringing together the various experts in a single virtual room? 1_2_3_4_5_6_7_8_9_10

Sb) Do you think that the META_EXP could improve the quality of surgical case discussion and preoperative planning thanks to the possibility of bringing together the various experts in a single virtual room? 1_2_3_4_5_6_7_8_9_10

Sc) How do you evaluate the possibility to have virtual lessons and surgical training in this virtual environment? 1_2_3_4_5_6_7_8_9_10
